# Errors in preparation of insulin infusions for critically ill patients

**DOI:** 10.1186/cc9811

**Published:** 2011-03-11

**Authors:** R Pierson, I Mackenzie

**Affiliations:** 1 Queen Elizabeth Hospital, Birmingham, UK

## Introduction

Dysglycaemia is associated with poorer outcomes in critically ill patients. Maintenance of normoglycaemia by the administration of intravenous insulin is an important therapy in the ICU, but many factors can affect plasma glucose levels in often unpredictable ways. Even if insulin could be delivered to patients at a guaranteed rate, the process of controlling glucose levels with exogenous insulin infusions is not straightforward. The preparation and administration of any drug for infusion is potentially subject to error. Insulin infusions are of particular concern, since they must be diluted from a concentrated stock solution. Random errors in the preparation of insulin infusions could result in significant differences between the concentration of insulin prescribed and that seen in the infusion. This would affect the rate of insulin delivery and could potentially result in unstable plasma glucose levels.

## Methods

Samples of 22 insulin infusions were taken over a 2-week period on a 14-bed adult general ICU. Each infusion had been prescribed as 1 IU/ml. After 10,000-fold dilution, samples were assayed using a two-step time-resolved fluorometric assay. To quantify the intra-assay variability, multiple aliquots were taken from a single sample of insulin. These were diluted and assayed in the same way as the ICU samples. Statistical analysis was performed via the SPSS computer package.

## Results

The 22 insulin solutions had a mean concentration of 0.99 IU/ml (SD 0.10, 95% CI: 0.95 to 1.03 IU/ml). The coefficient of variation was 10% (95% CI: 7.8 to 14.0%), with the insulin concentration ranging from 0.84 IU/ml to 1.16 IU/ml. Intra-assay coefficient of variation was found to be 3.6% (95% CI: 2.4 to 6.8%).

## Conclusions

The concentration of the insulin solutions studied varied from the prescribed concentration by up to 16%. This is probably due to random errors arising from differences in the methods of preparations of infusions by different nursing staff in the ICU. Insulin solutions could be prepared more accurately in a central location (for example, pharmacy), taking advantage of standardised techniques and equipment. This may reduce some of the random errors we have demonstrated and could potentially improve glycaemic control.

